# Patient Flow Dynamics in Hospital Systems During Times of COVID-19: Cox Proportional Hazard Regression Analysis

**DOI:** 10.3389/fpubh.2020.585850

**Published:** 2020-12-08

**Authors:** Sudhir Bhandari, Amit Tak, Sanjay Singhal, Jyotsna Shukla, Ajit Singh Shaktawat, Jitendra Gupta, Bhoopendra Patel, Shivankan Kakkar, Amitabh Dube, Sunita Dia, Mahendra Dia, Todd C. Wehner

**Affiliations:** ^1^Department of Medicine, S.M.S. Medical College & Attached Hospitals, Jaipur, India; ^2^Department of Physiology, S.M.S. Medical College & Attached Hospitals, Jaipur, India; ^3^Department of Physiology, Government Medical College, Barmer, India; ^4^Department of Pharmacology, S.M.S. Medical College & Attached Hospitals, Jaipur, India; ^5^Department of Rheumatology, Medstar Washington Hospital Center, Washington, DC, United States; ^6^Department of Horticultural Science, North Carolina State University, Raleigh, NC, United States

**Keywords:** COVID-19, cox proportion hazards models, evidence based decision making, hospital beds, public health, hospital management

## Abstract

**Objectives:** The present study is aimed at estimating patient flow dynamic parameters and requirement for hospital beds. Second, the effects of age and gender on parameters were evaluated.

**Patients and Methods:** In this retrospective cohort study, 987 COVID-19 patients were enrolled from SMS Medical College, Jaipur (Rajasthan, India). The survival analysis was carried out from February 29 through May 19, 2020, for two hazards: Hazard 1 was hospital discharge, and Hazard 2 was hospital death. The starting point for survival analysis of the two hazards was considered to be hospital admission. The survival curves were estimated and additional effects of age and gender were evaluated using Cox proportional hazard regression analysis.

**Results:** The Kaplan Meier estimates of lengths of hospital stay (median = 10 days, IQR = 5–15 days) and median survival rate (more than 60 days due to a large amount of censored data) were obtained. The Cox model for Hazard 1 showed no significant effect of age and gender on duration of hospital stay. Similarly, the Cox model 2 showed no significant difference of age and gender on survival rate. The case fatality rate of 8.1%, recovery rate of 78.8%, mortality rate of 0.10 per 100 person-days, and hospital admission rate of 0.35 per 100,000 person-days were estimated.

**Conclusion:** The study estimates hospital bed requirements based on median length of hospital stay and hospital admission rate. Furthermore, the study concludes there are no effects of age and gender on average length of hospital stay and no effects of age and gender on survival time in above-60 age groups.

## Key Messages

- Patient flow dynamic models are useful in management of the COVID-19 pandemic.
- Hospital data on admission and discharge can be used to estimate parameters of the model, such as hospital admission rates, recovery rates (inverse of median length of hospital stay).
- Real-time demand of hospital beds can be found based on estimated parameters.
- Evidence-based decision making is the best way to combat this pandemic.
- The intensity of public health measures implemented should be based on parameter values.

## Introduction

According to the World Health Organization, on June 22, 2020, there were 8,860,331 confirmed cases and 465,7440 deaths due to COVID-19 (1). The dynamics and course of COVID-19 are uncertain, and it is not merely possible but likely that the patient load will overwhelm the medical infrastructure, including hospital beds and medical equipment. The emergence of a pandemic leads to extraordinary demands on the public health system. The number of hospital beds occupied is a function of median length of hospital stay and admission rate (2). The public health measures during the management of a disease pandemic should be aimed at increasing hospital bed capacity and decreasing admission rates as well as the length of the median hospital stay. Currently, no pharmaceutical interventions are safe and effective; however, best practices for disease management are based primarily on non-pharmaceutical measures, including a ban on public gatherings, compulsory home stays, closure of religious and educational institutions, closure of non-essential businesses, face mask ordinances, quarantine, and *cordon sanitaire* (that is, a defined quarantine area from which those inside are not allowed to leave) (3). A number of mathematical models have been proposed to estimate the hospital bed capacity during the pandemic (4–6). The estimation of parameters is required for further analysis by such models.

The present study is an effort to estimate the dynamic parameters of the COVID-19 pandemic, including median length of hospital stay, median survival time, mortality rate, recovery rate, hospital admission rate, and case fatality rate in a tertiary care hospital. Further comparison of survival data across gender and age groups was performed using Cox proportional hazard analysis. Against the background of given parameters, the outcomes of public health policymaking can be evaluated. The rationale of evidence-based decision making can be fulfilled.

## Materials and Methods

In this hospital-based retrospective cohort study, 987 COVID-19 patients (confirmed with real-time RT-PCR) were enrolled from February 29 to May 19, 2020, from SMS Medical College and Hospital, Jaipur, Rajasthan, India. Survival analysis was carried out to estimate median hospital stay and median survival time. The effects of age and gender on survival patterns were evaluated using Cox proportional hazard regression analysis. Furthermore, case fatality, mortality, recovery, and hospital admission rates were also estimated. The duration of the study was 81 days.

### Data Collection

The age, gender, and dates of hospital admission and discharge were recorded from case sheets of patients. The hospital outcome, i.e., recovered, died, or admitted, was also recorded. Hazard 1 was considered to be hospital discharge or death. Survival time 1 (ST1) was calculated from a starting point as the date of hospital admission and an end point as the date of hospital discharge or death (Hazard 1). The cases admitted on the last day of the study were still considered under censored observations (censoring 1). Similarly, Hazard 2 was considered to be death in the hospital of patients over 60 years of age. Survival time 2 (ST2) was calculated as the period between the date of hospital admission (as all patients tested RT-PCR positive were hospitalized) and date of death (Hazard 2). The cases that were still admitted or recovered were considered under censored observations (censoring 2).

### Data Analysis

As the data was continuously observable, the survival analysis was done with the help of the Kaplan Meier (K-M) method. The survival rate was defined as a cumulative probability distribution function (cdf) of survival time (P[ST ≥ *t*], where *t* is time). Survival rates 1 (SR1) and 2 (SR2) for Hazards 1 and 2 were calculated.

In order to evaluate the effects of age and sex on survival patterns, two Cox proportional hazard models (Cox models 1 and 2) were fitted for Hazards 1 and 2, respectively. The covariates used in both models were age and gender. Before analyzing data in the Cox model, we checked to make sure censoring did not vary significantly for different values of covariates. The hazard ratios were calculated for both models (7).

The case fatality, mortality, recovery, and hospital admission rates were calculated as below (8):

$$\begin{array}{l} Case\;fatality\;rate\;\left(\%\right)=\\ \;\frac{Total\;number\;of\;deaths}{Total\;number\;of\;COVID-19\;cases}\times 100\\ Recovery\;rate\;\left(\%\right)=\\ \;\frac{Total\;number\;of\;recovered\;}{Total\;number\;of\;COVID-19\;cases}\times 100\\ Mortality\;rate\;\left(per\;100\;PD\right)=\\ \;\frac{Total\;number\;of\;deaths}{Total\;observed\;time\;\left(person-days\right)}\times 100\\ Hospital\;admission\;rate\;\left(per\;10^{5}\;PD\right)=\\ \;\frac{Total\;admissions}{population\times days}\times 10^{5} \end{array}$$

For the estimation of hospital admission rate, the population of Jaipur was considered to be 3.47 million (9).

### Statistical Analysis

The quantitative variables were expressed as median survival time and 95% confidence intervals with K-M based standard errors for the estimates of the Cox proportional hazard regression model. The statistical level of significance was considered at 5%. For the statistical analysis, we used JASP version 0.11 software and MATLAB 2016a (10, 11).

## Results

**Figure 1:**
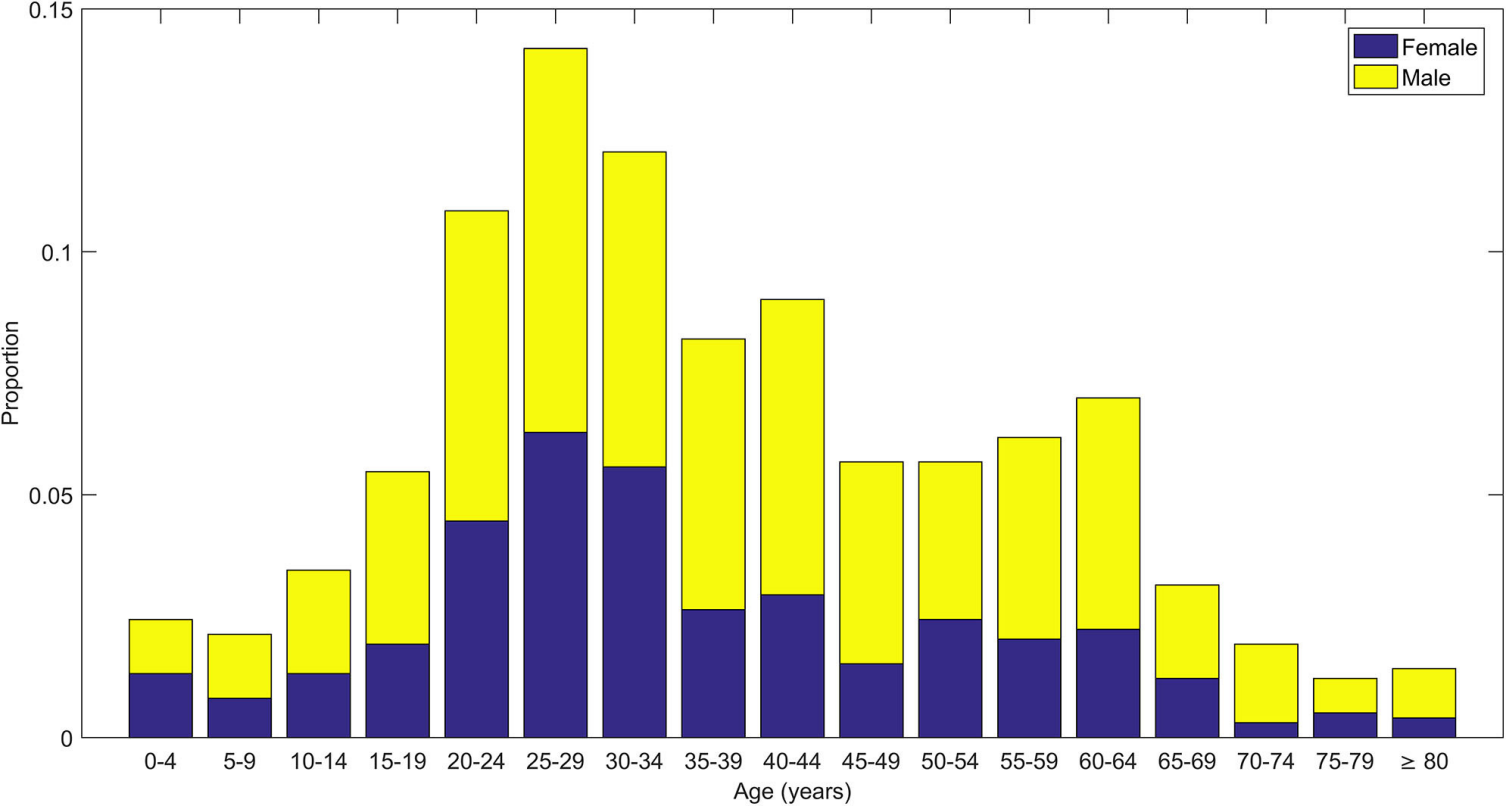
Stacked bar plots showing distribution of age of COVID-19 cases along with distribution of gender (male and female) within each age group.

The mean age of COVID-19 cases was 37.08 years (SD = 17.87). Men (62.11%) had a higher proportion of COVID-19 than women (37.89%). The distribution of age and gender indicated that younger men were most affected (Figure 1). The distribution of age and outcome showed a higher proportion of deaths in the elderly (Tables 1, 2).

**Table 1:** Descriptive statistics of age and sex along with comparison of age and gender across recovered and death cases in COVID-19 patients.

**Variables**		**Total (*N* = 987)**	**Recovered cases (*N* = 778)**	**Death cases (*N* = 80)**	Statistics^*^	**df^#^**	***p***
**Age**		34 (25, 50)	33 (24, 47.75)	55 (35, 65)	6.09	91	<0.001
	Male	62.11%	55.94%	5.94%			
**Sex**					0.13	1	0.72
	Female	37.89%	34.73%	33.79%		

* *Welch test was used to compare age in recovered and death cases and chi-squared test was used to find association between sex and cases*.

# *df: degrees of freedom; Median age and 1st and 3rd quartile is expressed in parenthesis*.

**Table 2:** Association of mortality and various age groups in COVID-19 patients.

**Age group (Years)**	**Mortality status**		
	**Death**	**Recovered**	**Total**
0-4	3	17	20
5–9	0	17	17
10–14	1	31	32
15–19	1	52	53
20–24	8	82	90
25–29	2	113	115
30–34	4	100	104
35–39	4	66	70
40–44	3	79	82
45–49	5	43	48
50–54	8	43	51
55–59	7	42	49
60–64	13	45	58
65–69	10	20	30
70–74	3	13	16
75–79	5	5	10
80	3	10	13
Total	80	778	858

### Survival Curves

**Figure 2:**
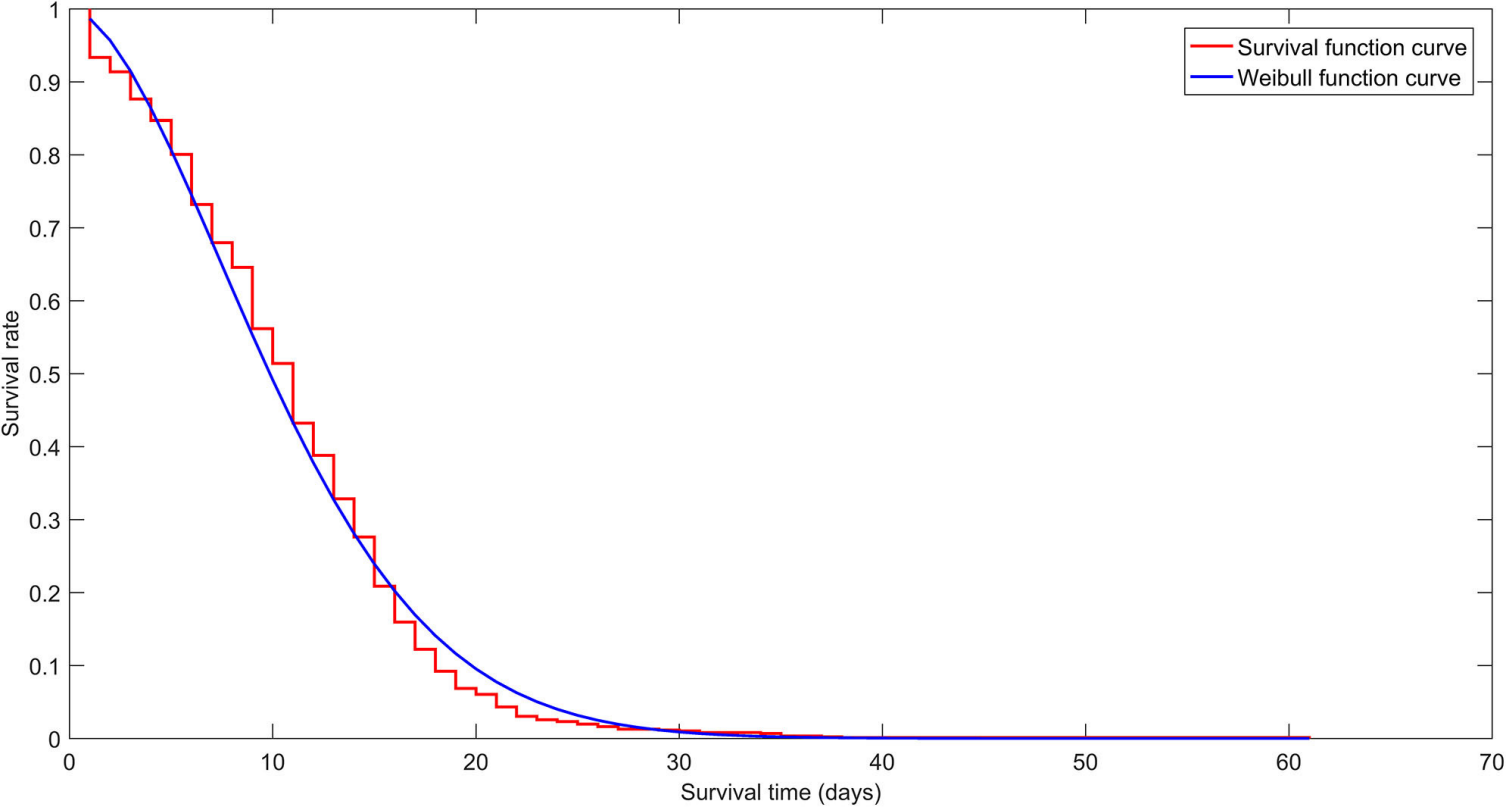
Survival curve (red staircase plot) for Hazard 1 shows Kaplan Meier estimates for all age groups. The Cox model was based on the Weibull function curve (blue line plot).

The survival curve and K-M estimates for Hazard 1 were obtained (Figure 2, Supplemental Table 1). The median ST1 (median hospital stay) was 10 days.

**Figure 3:**
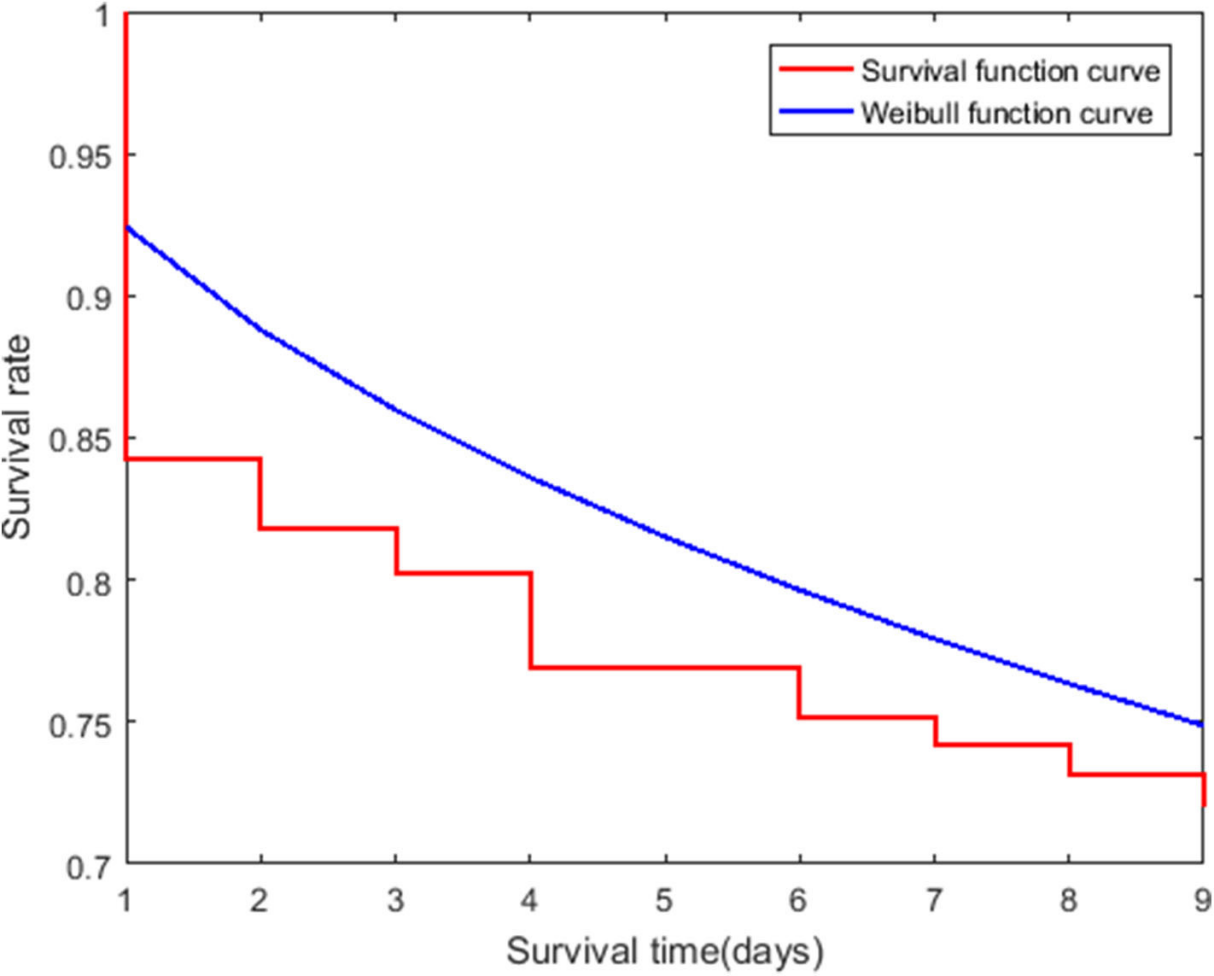
Survival curve (red staircase plot) for Hazard 2 shows Kaplan Meier estimates for patients over 60 years of age. The Cox model was based on the Weibull function curve (blue line plot).

The survival curve and K-M estimates for Hazard 2 were obtained (Figure 3, Supplementary Table 2). The median ST2 was more than 60 days because most of the data was censored.

### Cox Proportional Hazard Analysis

The censored and uncensored data for Hazard 1 did not differ significantly in mean age (*t* = 0.19, *p* = 0.85) and gender (χ^2^= 0.13, *p* = 0.71). Therefore, Cox model 1 was run with age and gender as covariates. Similarly, for Hazard 2, there was no significant difference found in mean age (*t* = 0.71, *p* = 0.48) and gender (χ^2^ = 0.26, *p* = 0.61). Therefore, Cox model 2 was also run with age and gender as a covariate.

The Cox Model 1 for SR1 showed no significant effect of age (HR = 1.00, *p* = 0.05) or gender (HR = 0.98, *p* = 0.88). Similarly, for SR2, the Cox model 2 showed no significant effects of age (HR = 1.01, *p* = 0.62) or gender (HR = 1.15, *p* = 0.69).

### Estimated Rates

The case fatality rate was estimated to be 8.1% (95% CI: 6.4–9.8%). The estimation of recovery rate was 78.8% (95% CI: 76.2–81.3%). The mortality rate was 0.10 (95% CI: 0.08–0.12) per 100 person-days, and the hospital admission rate was 0.35 (95% CI: 0.33–0.37) per 100,000 person-days.

## Discussion

The air we breathe, the food we eat, the house in which we live, the viruses to which we are exposed, the health services to which we have access, and the environment in which we live decide the outcome of a pandemic. The COVID-19 disease patterns are linked to migration, population movement, and disease diffusion (12). The main cause of varying rates of evolution of COVID-19 has resulted from different public health policies in various states (13). The primary objective for management of a pandemic is to keep the rate of evolution of cases lower such that the disease will not overwhelm the hospital bed capacity of any state. The aim of the management is to maintain the given inequality (2) (see Supplementary File for details):

$$\begin{array}{l} Hospital\;capacity\;of\;the\;system\;\geq median\;LOS\;\times HAR\;\times N\; \end{array}$$

where LOS is length of hospital stay, HAR is hospital admission rate (in 10^5^ person-days) and N is the population (10^5^ persons) dependent on the hospitals.

The present study estimated variables on the right side of the inequality. In order to maintain the inequality, hospital capacity should be increased or median hospital stay should be decreased or admission rate should be decreased. The hospital capacity of Jaipur was found to be 6,280, and the right side of the inequality was 108.5, which is less than hospital capacity (14). The rate of evolution for COVID-19 in Rajasthan was among the top eight states (15, 16).

As of now, no pharmaceutical agents are proven to be safe and effective for decreasing median hospital stay. The primary strategy is focused on non-pharmaceutical interventions (NPI) to decrease admission rates. Current control measures aim to reduce disease transmission through bans on public gatherings, compulsory home stays, closure of religious and educational institutions, closure of non-essential businesses, face mask ordinances, quarantine, and cordon sanitaire (that is, a defined quarantine area from which those inside are not allowed to leave) (3). Ravaghi et al. reviewed methods for determining optimum hospital capacity. The main factors were average length of hospital stay, admission rate, discharge rate, and target bed occupancy rate (2).

A number of mathematical models have been used in the prediction of hospital beds during the pandemic. Some are data-driven models as used by Manca et al. for the prediction of ICU beds (6). Others are empirical models, including SIR, SIRD, SEIR and SEIRD, and SIDARTHE (4). A number of models were proposed for estimating hospital bed capacity based on queuing theory. Patient demand for beds was modeled with Poisson distribution with rate λ. The service duration has an exponential distribution 1/μ (5). Further analysis of the model requires parameters like λ and μ. The present study estimates parameters for further analysis of such models.

One approach to decrease the median length of hospital stays is to triage patients based on requirement of specialized care with beds allotted accordingly. The National Institute of Health and Care Excellence (NICE) has published an algorithm to ensure appropriate admissions to the ICU for those most in need (17). In a study of the prediction of length of hospital stay with liver blood test results, liver condition (HBsAb positive, HBcAb positive, and fatty liver disease) was carried out. The median length of hospital stay was 6 days (18). Bhandari mentioned differential neutrophil count and random blood sugar as predictors of mortality risk of COVID-19 (19). One study reported that BMI, age, and CRP were all related to prolongation of length of hospital stay (20). Factors responsible for prolonged LOS in which the median was 11 days (IQR, 5–15 days) showed the most important were lower neutrophil counts, higher partial thrombin time (PT), lower D-Dimer associated with prolonged length of stay at hospital (21). A novel strategy to manage patients is to triage based on disease severity with management of mild patients in shelter homes. The shelter homes are large-scale, temporary hospitals, assembled rapidly by converting existing public places such as stadiums and exhibition centers into healthcare facilities. The important characteristics of shelter homes are rapid construction, large scale, and low cost. They serve functions of isolation, basic medical care, triage, frequent monitoring and referral, essential living, and social engagement (22).

Finally, the WHO Scientific and Technical Advisory Group for Infectious Hazards (STAG-IH) reviewed available information about COVID-19 and focused on closure monitoring of epidemiology, communication strategies, intensive source control, continued containment activities, intensified active surveillance, resilience of health systems, mitigation activities during community transmission, development of serological tests, and continued research (23).

### Conclusion

The present study will help facilitate an evidence-based decision-making process for management of the COVID-19 pandemic. The estimation of dynamic parameters of patient flow in a hospital helps in hospital management. Further, the parameters can be used by various mathematical models to predict future requirements.

### Limitations of the Study

The study includes only age and gender as covariates to run the model. The clinical covariates, such as severity, symptoms, and CT scores may provide more precise information about survival time and length of hospital stay.

## Data Availability Statement

The data analyzed in this study is subject to the following licenses/restrictions: Data is available on reasonable request to corresponding author. Requests to access these datasets should be directed to Amit Tak, dramittak@gmail.com.

## Ethics Statement

The studies involving human participants were reviewed and approved by Ethics Committee, SMS Medical College, Jaipur (Letter No. 524/MC/EC/2020 dated 7 July 2020). Written informed consent for participation was not provided by the participants' legal guardians/next of kin because: Ethics Committee said, as per the National Ethical Guidelines for Biomedical and Health Research involving Human Participants by Indian Council of Medical Research, 2017 (section 5: Informed Consent Process- Box 5.2, Page No 53 and 54), the study being retrospective where participants have been deidentified, the waiver of informed consent is hereby granted.

## Author Contributions

SB, AD, and JS provided administrative support, AT, SD, MD, and TW did concept, design, and data analysis and interpretation. AS helped in provision of patients. SK helped in collection and assembly of data. BP, SS, and JG helped in manuscript writing. All authors commented and finally approved the manuscript.

## Conflict of Interest

The authors declare that the research was conducted in the absence of any commercial or financial relationships that could be construed as a potential conflict of interest.

## Abbreviations

cdf: cumulative probability distribution function
CI: confidence interval
COVID-19: coronavirus disease-19
K-M: Kaplan Meier
HR: hazard ratio
*p*: *p*-value
SARS CoV-2: severe acute respiratory corona virus 2
SR1: survival rate 1
SR2: survival rate 2
ST1: survival time 1
ST2: survival time 2.

## References

[1] World Health Organization Coronavirus Disease- 2019. (2020). Availabe online at: https://www.who.int/emergencies/diseases/novel-coronavirus-2019 (accessed June 18, 2020).

[2] Ravaghi H Alidoost S Mannion R Bélorgeot VD. Models and methods for determining the optimal number of beds in hospitals and regions: a systematic scoping review. BMC Health Serv Res. (2020) 20:186. 10.1186/s12913-020-5023-z 32143700 PMC7060560

[3] Hartley DM Perencevich EN. Public health interventions for COVID-19. JAMA. (2020) 323:1908. 10.1001/jama.2020.5910 32275299

[4] Giordano G Blanchini F Bruno R Colaneri P Di Filippo A Di Matteo A. Modelling the COVID-19 epidemic and implementation of population-wide interventions in Italy. Nat Med. (2020) 26:855–60. 10.1038/s41591-020-0883-7 32322102 PMC7175834

[5] Green LV. How many hospital beds? INQUIRY. (2002) 39:400–12. 10.5034/inquiryjrnl_39.4.400 12638714

[6] Manca D Caldiroli D Storti E. A simplified math approach to predict ICU beds and mortality rate for hospital emergency planning under Covid-19 pandemic. Comput Chem Eng. (2020) 140:106945. 10.1016/j.compchemeng.2020.106945 32565584 PMC7271874

[7] Indrayan A Malhotra RK. Medical Biostatistics. in Survival Analysis. 4th ed. Boca Raton, FL: CRC Press, Taylor & Francis Group (2018). p. 513–23.

[8] Epidemiology Working Group for NCIP Epidemic Response Chinese Center for Disease Control and Prevention. [The epidemiological characteristics of an outbreak of 2019 novel coronavirus diseases (COVID-19) in China]. Zhonghua Liu Xing Bing Xue Za Zhi. (2020). 41:145–51. 10.3760/cma.j.issn.0254-6450.2020.02.003 32064853

[9] Jaipur Rajasthan Official Population. (2020). Available online at: https://jaipur.rajasthan.gov.in/content/raj/jaipur/en/about-jaipur/population.html# (accessed June 25, 2020).

[10] JASP Team JASP version 0.12.2 [Computer software]. Amsterdam: University of Amsterdam, Copyright 2013-2019

[11] MATLAB Team Statistics Machine Learning Toolbox 10.2, Classification Learner App. MATLAB. version 9.0.0.341360 (R 2016a). Natick, MA: The Mathworks Inc (2016).

[12] Dummer TJB. Health geography: supporting public health policy and planning. Canad Med Assoc J. (2008) 178:1177–80. 10.1503/cmaj.071783 18427094 PMC2292766

[13] Bhandari S Shaktawat AS Tak A Patel B Gupta K Gupta J A multistate ecological study comparing evolution of cumulative cases (trends) in top eight COVID-19 hit Indian states with regression modeling. Int J Acad Med. (2020) 6:91–5. 10.4103/IJAM.IJAM_60_20

[14] Government of Rajasthan Department of Medical, Health and Family Welfare. (2020). Available online at: http://www.rajswasthya.nic.in/PDF/COvid%20Facility%20Rajasthan.pdf (accessed June 23, 2020).

[15] Bhandari S Sharma R Singh Shaktawat A Banerjee S Patel B Tak A COVID-19 related mortality profile at a tertiary care centre: a descriptive study. Scr Med. (2020) 51:69–73. 10.5937/scriptamed51-27126

[16] Kakkar S Bhandari S Shaktawat A Sharma R Dube A Banerjee S. A preliminary clinico-epidemiological portrayal of COVID-19 pandemic at a premier medical institution of North India. Ann Thoracic Med. (2020) 15:146. 10.4103/atm.ATM_182_20 32831936 PMC7423199

[17] Nicola M O'Neill N Sohrabi C Khan M Agha M Agha R. Evidence based management guideline for the COVID-19 pandemic - Review article. Int J Surg. (2020) 77:206–16. 10.1016/j.ijsu.2020.04.001 32289472 PMC7151371

[18] Gu X Li X An X Yang S Wu S Yang X. Elevated serum aspartate aminotransferase level identifies patients with coronavirus disease 2019 and predicts the length of hospital stay. J Clin Lab Anal. (2020) 34:e23391. 10.1002/jcla.23391 32488888 PMC7300531

[19] Bhandari S Shaktawat AS Tak A Patel B Shukla J Singhal S Logistic regression analysis to predict mortality risk in COVID-19 patients from routine hematologic parameters. Ibnosina J Med Biomed Sci. (2020) 12:123–9. 10.4103/ijmbs.ijmbs_58_20

[20] Moriconi D Masi S Rebelos E Virdis A Manca ML De Marco S. Obesity prolongs the hospital stay in patients affected by COVID-19, and may impact on SARS-COV-2 shedding. Obesity Res Clin Pract. (2020) 14:205–9. 10.1016/j.orcp.2020.05.009 32534848 PMC7269944

[21] Hong Y Wu X Qu J Gao Y Chen H Zhang Z. Clinical characteristics of Coronavirus Disease 2019 and development of a prediction model for prolonged hospital length of stay. Annal Trans Med. (2020) 8:443. 10.21037/atm.2020.03.147 32395487 PMC7210129

[22] Chen S Zhang Z Yang J Wang J Zhai X Bärnighausen T. Fangcang shelter hospitals: a novel concept for responding to public health emergencies. Lancet. (2020) 395:1305–14. 10.1016/S0140-6736(20)30744-3 32247320 PMC7270591

[23] Heymann DL Shindo N. COVID-19: what is next for public health? Lancet. (2020) 395:542–5. 10.1016/S0140-6736(20)30374-3 32061313 PMC7138015

