# Supplementary material for: Patient Flow Dynamics in Hospital Systems During Times of COVID-19: Cox Proportional Hazard Regression Analysis
Source: Front Public Health. 2020 Dec 8;8:585850. doi: 10.3389/fpubh.2020.585850 (PMC7793894; doi:10.3389/fpubh.2020.585850)
Supplement: Supplementary file 1 [file Data_Sheet_1.PDF]

Patient flow dynamics in hospital systems during times of COVID-19: Cox proportional hazard regression analysis.

Dynamic model of hospital bed occupancy

Let the number of beds occupied in short period of time is proportional to the hospital admission rate and number of beds vacant in short period of time is proportional to recovery rate times the number of beds occupied. It can be shown that at any point of time the rate of change of number of occupied beds is :

$$\frac{dB}{dt} = HAR \times N - RR \times B$$

Where B is number of beds occupied , HAR is the hospital admission rate, N is the population of patients dependent on health system, RR is the recovery rate. The recovery rate is inverse of median length of hospital stay.

At equilibrium ,

$$\frac{dB}{dt} = 0$$

$$B_{eq} = \frac{HAR \times N}{RR} = HAR \times N \times median\ LOS$$

where B_{eq} is the hospital capacity of any system and LOS is length of hospital stay. When B_{eq} exceeds the hospital capacity of the system there is acute shortage of hospital beds. So decisions should be focused either on increase the hospital capacity of the system or decrease in the hospital admission rate such that

$$Hospital\ capacity\ of\ the\ system\ \geq median\ LOS\ \times HAR\ \times N$$

Supplementary Information File – contains two tables.

Table Legends

Table S1. shows Kaplan Meier estimates after survival analysis for ‘Hazard 1’(i.e., hospital discharge) with 95% confidence intervals.

Table S2 shows Kaplan Meier estimates after survival analysis for ‘Hazard 2’(i.e., hospital death) with 95% confidence intervals.

Table S1. shows Kaplan Meier estimates after survival analysis for ‘Hazard 1’(i.e., hospital discharge) with 95% confidence intervals.

ST1 (days)	SR1 (cdf)	LL	UL
1	1		
1	0.934	0.917	0.950
2	0.914	0.895	0.933
3	0.876	0.854	0.898
4	0.847	0.823	0.871
5	0.801	0.774	0.827
6	0.732	0.702	0.762
7	0.679	0.648	0.711
8	0.646	0.614	0.678
9	0.562	0.529	0.595
10	0.514	0.481	0.547
11	0.432	0.399	0.466
12	0.388	0.356	0.421
13	0.329	0.297	0.360
14	0.276	0.246	0.306
15	0.209	0.181	0.236
16	0.160	0.135	0.184
17	0.122	0.100	0.144
18	0.092	0.073	0.111
19	0.069	0.052	0.086
20	0.061	0.045	0.077
21	0.043	0.030	0.057
22	0.030	0.019	0.042
23	0.026	0.015	0.036
24	0.023	0.013	0.033
25	0.020	0.010	0.029
26	0.016	0.008	0.025
27	0.013	0.005	0.020
29	0.012	0.004	0.019
30	0.010	0.004	0.017
31	0.008	0.002	0.014
34	0.007	0.001	0.013
35	0.003	0.000	0.007
37	0.002	0.000	0.006
38	0.001	0.000	0.003
61	0.000		

*ST1: survival time 1; SR1: survival rate 2; cdf : cumulative probability distribution function; CI: confidence interval; LL: lower limit of CI; UL: upper limit of CI

Table S2 shows Kaplan Meier estimates after survival analysis for ‘Hazard 2’(i.e., hospital death) with 95% confidence intervals in patients above 60 years of age.

		95% CI	
ST2 (days)	SR2 (cdf)	LL	UL
1	1.0000		
1	0.8425	0.7792	0.9059
2	0.8184	0.7513	0.8856
3	0.8024	0.7330	0.8718
4	0.7693	0.6956	0.8430
6	0.7516	0.6756	0.8276
7	0.7421	0.6648	0.8194
8	0.7315	0.6526	0.8104
9	0.7201	0.6393	0.8009

*ST2: survival time 2; SR2: survival rate 2; cdf : cumulative probability distribution function; CI: confidence interval; LL: lower limit of CI; UL: upper limit of CI
